# Selective Oxidation of Polysulfide Latexes to Produce Polysulfoxide and Polysulfone in a Waterborne Environment

**DOI:** 10.1021/acs.macromol.1c00382

**Published:** 2021-04-08

**Authors:** Lorena Infante Teixeira, Katharina Landfester, Héloïse Thérien-Aubin

**Affiliations:** Max Planck Institute for Polymer Research, Ackermannweg 10, 55128 Mainz, Germany

## Abstract

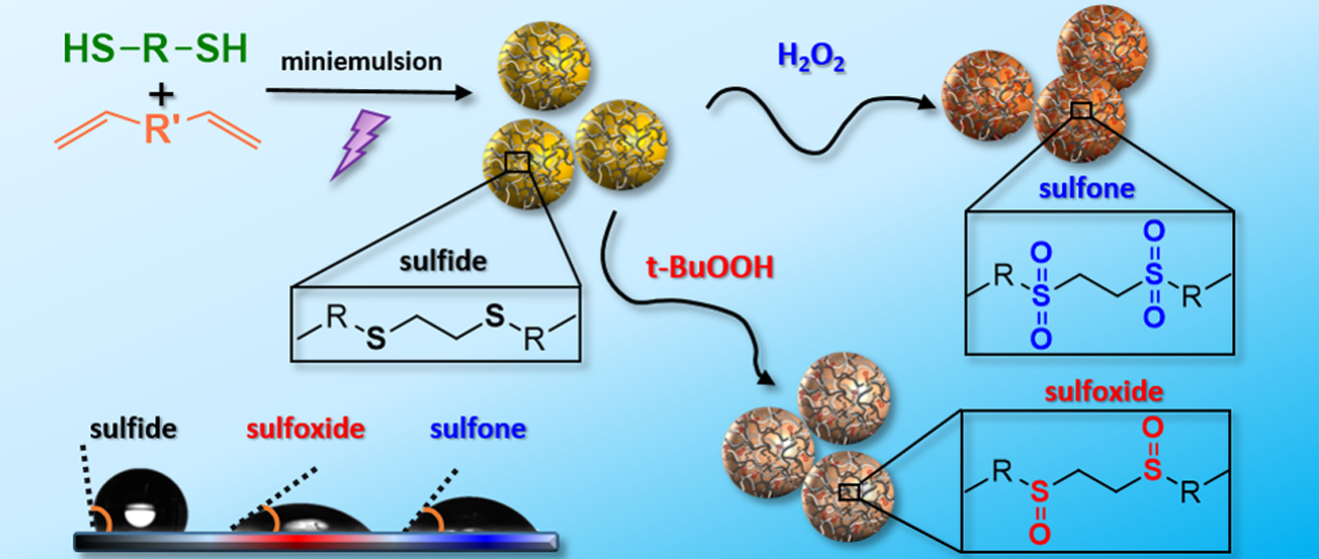

Polymers containing sulfur centers with high oxidation states in the main chain, polysulfoxide and polysulfone, display desirable properties such as thermomechanical and chemical stability. To circumvent their challenging direct synthesis, methods based on the oxidation of a parent polysulfide have been developed but are plagued by uncontrolled reactions, leading either to ill-defined mixtures of polysulfoxides and polysulfones or to polysulfones with reduced degrees of polymerization due to overoxidation of the polymer. We developed an alternative method to produce well-defined polysulfoxide and polysulfone in a waterborne colloidal emulsion using different oxidants to control the oxidation state of sulfur in the final materials. The direct oxidation of water-based polysulfide latexes avoided the use of volatile organic solvents and allowed for the control of the oxidation state of the sulfur atoms. Oxidation of parent polysulfides by *tert*-butyl hydroperoxide led to the production of pure polysulfoxides, even after 70 days of reaction time. Additionally, hydrogen peroxide produced both species through the course of the reaction but yielded fully converted polysulfones after 24 h. By employing mild oxidants, our approach controlled the oxidation state of the sulfur atoms in the final sulfur-containing polymer and prevented any overoxidation, thus ensuring the integrity of the polymer chains and colloidal stability of the system. We also verified the selectivity, versatility, and robustness of the method by applying it to polysulfides of different chemical compositions and structures. The universality demonstrated by this method makes it a powerful yet simple platform for the design of sulfur-containing polymers and nanoparticles.

## Introduction

Sulfur-containing polymers are a class of high-performance materials and have been instrumental in the development of specialty applications. While polysulfide (PSR) can be prepared directly from polycondensation,^1,2^ the direct synthesis of polysulfoxide (PSO) and polysulfone (PSO2) is challenging.^3,4^ However, the polymers containing sulfur centers with higher oxidation states, polysulfoxide (PSO) and polysulfone (PSO2), display desirable properties, such as thermomechanical and chemical stability.^5,6^ Other polymers such as those containing phosphorous are also good examples of how the oxidation state of the main chain heteroatom can influence the properties of the polymer.^7^

To circumvent the synthetic challenge involved in the direct production of PSOs and PSO2s, for example, by Friedel–Crafts polysulfonylation process or a nucleophilic substitution of activated aromatic halides,^8−10^ alternative methods based on the oxidation of a parent PSR have been developed.^11,12^ This strategy leads to a second drawback: the oxidation is often plagued by uncontrolled reactions, leading to the coexistence of PSO and PSO2 or the depolymerization of the chains due to the overoxidation of the sulfur centers.^13,14^ However, it is of critical importance to produce these oxidized derivatives as pure species to understand and harness the full potential of these PSOs and PSO2s. This critical challenge has been tackled using different approaches, and the selective oxidation of polysulfide materials to either PSO or PSO2 by controlling the oxidation conditions has been achieved for selected polysulfides.^11,15,16^ For example, methods based on a delicate balance of the ratio between oxidant and sulfur(II) center have been successful in producing pure oxidized species.^15,16^ Alternative methodologies, such as selective oxidation based on the use of a selenium catalyst, could also be a solution.^17^ However, most of these strategies require the use of organic solvents and are incompatible with some polymer systems. To address these limitations, we need to develop an approach based on the use of mild oxidation conditions to yield pure PSOs and PSO2s in polysulfide latex suspensions.

The synthesis of PSO and PSO2 by the oxidation of a parent PSR creates a platform that enables chemical modification of the polymer chains.^18^ Such an approach offers a simple and straightforward synthetic method, with unmatched potential in structural versatility as it solely relies on the previously synthesized PSR network. Furthermore, the use of thiol-ene polymerization allows for the production of a broad range of parent PSRs, bearing various functional groups using suitable monomers.^14,19,20^ Previous studies have demonstrated the convenience of the oxidation of the parent PSR in different oxidative environments, reporting the synthesis of PSO2s or mixtures of both PSO and PSO2 from a parent PSR in solution, in bulk, or following the immersion of PSR films or three-dimensional (3D) objects.^12,14,21,22^ Although these processes yield high-oxidation-state sulfur-containing polymers, they remain deficient for the production of high-quality PSO and PSO2 in terms of processability and the use of volatile organic solvents but mostly in the poor quantitative control of the composition of either pure PSO or PSO2.

Alternatively, it is possible to use water-based dispersions of PSR as the starting material.^11,13,18^ From a practical standpoint, the oxidation conditions required for the formation of PSO and PSO2 from a parent PSR are compatible with water-based suspensions, due to the hydrophilic nature of commonly used oxidants. Also, such a method could offer improved processability by circumventing the excessive use of organic solvents and the production of toxic volatile organic compounds (VOCs) common to other approaches^12,22^ and by enabling the use of high-solid-content suspensions without significant variation of the viscosity of the system. Consequently, it is highly beneficial for the large-scale production of PSO and PSO2. However, from the chemical point of view, uncontrolled oxidation or overoxidation usually follows the synthesis of oxidized derivatives of PSRs, regardless of the medium in which it occurs. Therefore, the resulting oxidized sulfur-containing polymers are either ill-defined mixtures of PSOs and PSO2s or pure PSO2s with reduced molecular weight due to chain scission.^13,23^

To address these issues, a milder and more selective synthetic method is necessary. We propose an approach based on the controlled oxidation of parent PSRs using mild oxidants, such as hydrogen peroxide (H_2_O_2_) and *tert*-butyl peroxide (*t*-BuOOH) in an aqueous environment, to control the oxidation state of the sulfur atoms in the final polymer. The oxidation reaction occurs directly in a waterborne suspension, avoiding the use of volatile organic solvents during the reaction, as well as taking advantage of the high surface-to-volume ratio of the nanoparticles (NPs) in suspension for improved reaction kinetics when compared to the same reaction in bulk.^11,13^ By employing mild oxidants, our approach provides control over the oxidation state of the final sulfur-containing derivative and prevents any overoxidation, thus ensuring the integrity of the polymer backbone. Due to this newly gained control, polymers with intermediate oxidation states, PSO, can be synthesized as pure compounds and exploited for potential applications. Here, we also show that the control over the degree of oxidation of the sulfur centers provides a way to tune the properties of the sulfur-containing polymers, such as hydrophilicity and glass transition temperature. Our approach provides unmatched potential in developing a new VOC-free platform for polymer design with on-demand properties with the simplicity of a one-pot process.

## Experimental Section

### Materials

Diallyl adipate (DAA, 98%), triallyl 1,3,5-benzenetricarboxylate (TAP, 98%), (+)-limonene (LIM, 95%), hexadecane (>99.5%), and *tert*-butyl hydroperoxide (*t*-BuOOH, 70% solution) were purchased from TCI Deutschland. Diallyl phthalate (DAP, 98%), divinyl benzene (DVB, 70%), sodium dodecyl sulfate (SDS, >99%), tetrahydrofuran (THF, 99.9%), dimethylformamide (DMF, 99%), and chloroform-*d* (CDCl_3_, 99.8%) were acquired from Sigma-Aldrich. 2,2′-(Ethylenedioxy) diethanethiol (EDDT, 95%) was obtained from Bruno Bock, 1,4-benzenedithiol (1,4DTB, 97%) from Alfa-Aesar, Irgacure 2959 (I2959) from BASF, 2,2,2-trifluoroacetophenone (TFAP, 99%) from Acros Organics, and hydrogen peroxide solution (H_2_O_2_, 30%) from Merck. All chemicals were used as received unless noted otherwise. Divinyl benzene was purified prior to use with a column of aluminum oxide.

### Characterization

Nuclear magnetic resonance, ^13^C NMR and ^1^H NMR, spectra were recorded in deuterated chloroform, unless noted otherwise, on a Bruker Avance 300 MHz spectrometer. Fourier-transform infrared (FTIR) measurements were recorded with a Perkin Elmer Spectrum BX spectrometer in ATR mode. Thermogravimetric analyses (TGA) were conducted under a N_2_ atmosphere between 25 and 600 °C at a rate of 10 °C·min^–1^ on a Mettler-Toledo TGA/SDTA-851 thermobalance. Three cycles of differential scanning calorimetry (DSC) were performed between −140 and +250 °C at a heating/cooling rate of 10 K min^–1^ on a 204F1/ASC Phoenix calorimeter. Gel permeation chromatography (GPC) was performed on a PSS Security of Agilent Technologies 1260 Infinity with THF or DMF as the mobile phase. The GPC was calibrated with a series of poly(methyl methacrylate) (PMMA). A Malvern Zetasizer Nano-S90 dynamic light scattering (DLS) instrument was used to measure the hydrodynamic diameter and size distribution of the nanoparticles in dilute suspensions. The water contact angles were measured on films of polymer spin-casted on glass with a DataPhysics OCA35 telescopic goniometer.

### Synthesis of Nanoparticles of Polysulfide in a Dispersed Medium

The polysulfide nanoparticles were prepared by photoinitiated thiol-ene polymerization of a monomer mixture in a miniemulsion. In a typical reaction, a biphasic mixture of a dispersed phase composed of the monomer equimolar mixture (*C*_monomers_ = 20 wt % to the emulsion), containing hexadecane (4 wt % of *C*_monomers_), a continuous phase composed of water, SDS as a surfactant (*C*_SDS_ = 0.2 wt % in water), and the photoinitiator I2959 (3 wt % of *C*_monomers_), was emulsified by ultrasonication in a Branson digital sonifier 450 cell disruptor (70% amplitude, 2 min). The resulting miniemulsion was placed in a thin-wall quartz tube inside a UV-reactor and irradiated for 4 h at 385 nm under continuous magnetic stirring. The conversion of the monomers measured by ^1^H NMR spectroscopy in CDCl_3_ from the consumption of the allyl (or vinyl) protons indicated the completion of the reaction.

### Oxidation of Polysulfide Nanoparticles in a Dispersed Medium

The oxidation of the suspension of polysulfide nanoparticles (NPs) was performed by mixing 5 g of a suspension containing 5 wt %. of NP (or ca. 1 mmol of S(II)) with a 10-fold excess of the oxidant in comparison to the concentration of the S(II) center in the suspension of nanoparticles (10.7 mmol of the oxidant: 1.08 mL of H_2_O_2_ or 1.47 mL of *t*-BuOOH), followed by the addition of TFAP (50 μL, 0.352 mmol). After a predetermined reaction time, a 2 mL aliquot of the reaction mixture was poured in a centrifuge tube containing 10 mL of brine, followed by centrifugation (20 kG, 8 min, 5 °C). The oxidized NPs were cleaned further by redispersion in 10 mL of aqueous SDS solution (5–6 wt % NP), followed by precipitation in brine and centrifugation (2×). The final NPs were redispersed in distilled water.

A fraction of the purified suspension was then dried in an oven at 65 °C under reduced pressure overnight. Then, ca. 500 mg of the polymer was dissolved in 1 mL of THF and precipitated in 10 mL of water or brine under stirring, and the polymer was recovered by centrifugation (20 kG, 8 min, 5 °C) (2×). The purified polymers were then dried overnight in a vacuum oven and used for FTIR, ^13^C NMR, and contact angle analyses. To prepare the films used for contact angle measurements, ca. 50 mg of the dried samples was dissolved in 500 μL of CHCl_3_ or DMF. These polymer solutions were spin-coated on clean glass slides and annealed overnight at 80 °C.

### Kinetics of Oxidation and the Oxidation State

To measure the extent of oxidation, FTIR spectra were recorded at different intervals of time during the course of the reaction. The peak at ca. 1030 cm^–1^ corresponds to a stretching of the S=O bond in sulfoxides, and the peak at ca. 1122 cm^–1^ resulting from the stretching of O=S=O was used to monitor the reaction. During the quantification of each species, the integral of the relevant peak was normalized against a peak not involved in the reaction, either the C=O stretching at 1728 cm^–1^ for the polymers containing ester linkages or the peak of the C–H stretching at 2890 cm^–1^ for the other polymers.

The composition of the polymer was also quantified by ^13^C NMR spectroscopy. Quantitative ^13^C spectra were recorded using an inverse-gated pulse sequence. The signals from the C atoms close to the sulfur in α and β positions were chosen as markers of the oxidation state. The chemical shifts of those carbon atoms at 70.9, 29.2, and 28.8 ppm (sulfides); 63.5, 52.5, and 49.8 ppm (sulfoxides); and 53.3, 64.7, and 51.6 ppm (sulfones) enabled the quantification of each species throughout the course of the reaction.

## Results and Discussion

Miniemulsion photoinitiated thiol-ene polymerization of five PSR NPs was carried out to address the structure–property relationship of the final NPs and their oxidized derivatives using different monomer mixtures. The different thiol-ene couples investigated were 2,2′-(ethylenedioxy) diethanethiol with diallyl adipate (EDDT-DAA), EDDT with diallyl phthalate (EDDT-DAP), EDDT with limonene (EDDT-LIM), 1,4-dithiol benzene with divinyl benzene (1,4DTB-DVB), and 1,3-dithiol benzene with divinyl benzene (1,3DTB-DVB). Following the irradiation of the miniemulsion with UV light at 385 nm, ^1^H NMR spectroscopy was used to quantify the conversion of the allyl or vinyl protons of the ene (Table S1). The characterization of the suspension and the resulting polymers was performed with a combination of GPC, DLS, Scanning electronic microscopy (SEM), TGA, and DSC.

The colloidal properties of the PSR NPs (size and polydispersity index) were largely independent of the monomer mixture used, while the polymer chains composing the PSR NPs displayed distinct properties. The number-average molecular weight (*M*_n_) of the polymer prepared varied significantly for the different thiol-ene couples (Table S1). The final *M*_n_ of the PSR polymers was highly dependent on the degree of conversion of the monomers, a direct consequence of the step-growth mechanism of the thiol-ene polycondensation reaction. The conversion was more limited for the less reactive dienes,^24,25^ such as limonene. Additionally, certain dithiols, like dithiol benzene, contained up to 4% of impurities in the form of monothiol derivatives influencing the stoichiometry of the reaction and the extent of conversion of the monomers. These thiol-ene couples resulted in lower conversions, as measured by NMR spectroscopy, and yielded polymers with lower *M*_n_.

**Figure 1:**
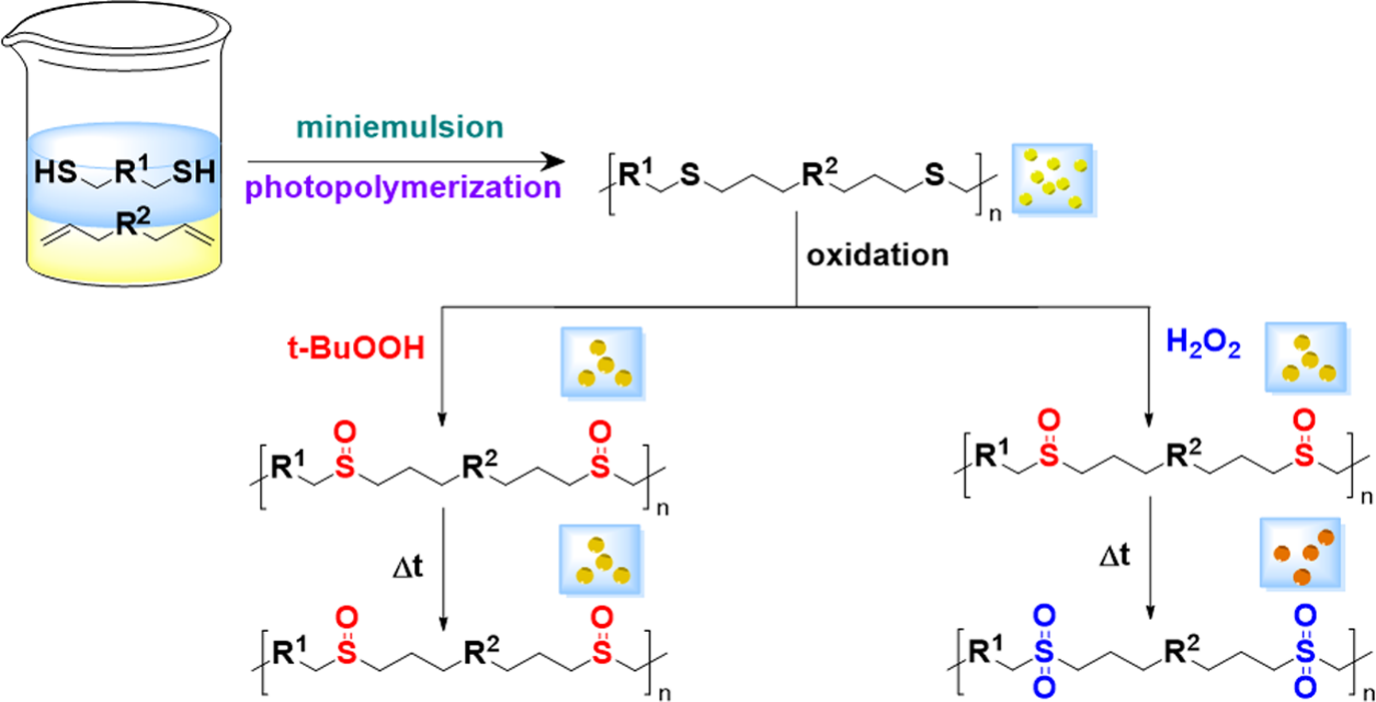
Scheme of the synthesis and oxidation of polysulfide nanoparticles in water-based suspension. Different oxidants and reaction conditions can yield nanoparticles with different degrees of oxidation.

**Figure 2:**
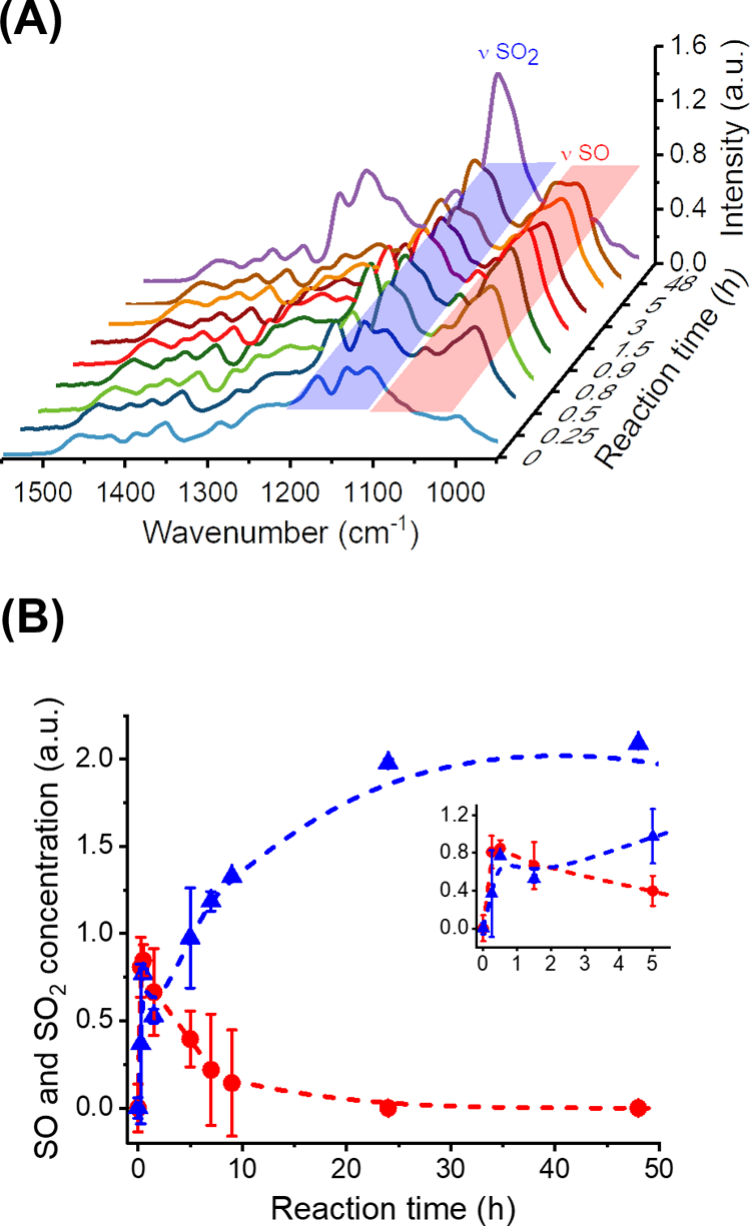
Oxidation kinetics of polysulfide NPs by H_2_O_2_ measured by FTIR spectroscopy. (A) FTIR spectra over time, showing the appearance of both SO and SO_2_ species. (B) Relative concentration of SO (red) and SO_2_ (blue) in the polymer nanoparticles. The inset shows the initial 5 h of the reaction kinetics.

The conversion of these parent PSR NP suspensions, through oxidation, yielded the targeted polysulfone (PSO2) and polysulfoxide (PSO) NPs (Figure 1). The oxidation of sulfur-containing polymer NPs has been used to trigger the release of payloads.^23,26−29^ However, such an approach relies on the overoxidation of the sulfur centers to initiate the degradation of the polymer network leading to the release of the encapsulated payload but has been incompatible in isolating pure PSO and PSO2 NPs. Consequently, one of the main challenges faced when using oxidants in the presence of PSR to PSO or PSO2 is the uncontrolled oxidation leading to the formation of a mixture of PSR, PSO, and PSO2 or even to the complete degradation of the main chain due to the overoxidation of the sulfur center in the backbone.^13,30^ To gain more control over the oxidation reaction, we analyzed the kinetics of the conversion of aqueous suspensions of PSR NPs in the presence of low concentrations of two different oxidizing agents (Figure 2).

**Figure 3:**
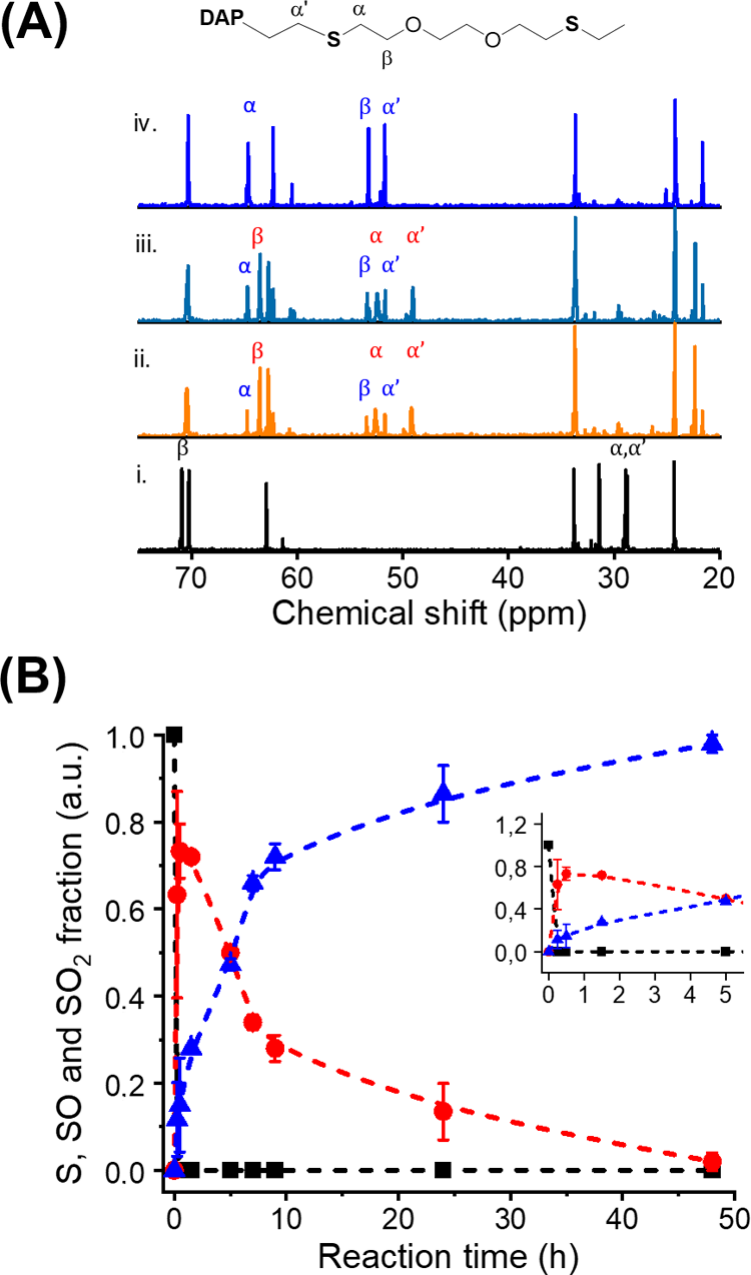
Oxidation kinetics of polysulfide NPs by H_2_O_2_ measured by ^13^C NMR spectroscopy. (A) ^13^C NMR spectra of the polymer NPs after (i) 0 h, (ii) 5 h, (iii) 10 h, and (iv) 24 h of oxidation, showing the formation of SO (red) and SO_2_ (blue) species and the disappearance of the parent sulfide (black). (B) Concentration of S (black), SO (red), and SO_2_ (blue) in the polymer nanoparticles. The inset shows the initial 5 h of the reaction kinetics.

The NPs prepared with EDDT-DAA were used as a model PSR to study the different oxidation conditions. The kinetics of the oxidation with H_2_O_2_ was monitored by FTIR and ^13^C NMR spectroscopies (Figures 2 and 3). H_2_O_2_-mediated oxidation is known to promote a stepwise type of oxidation of the sulfur centers, going from sulfur(II) to sulfur(IV) in a matter of minutes and then to sulfur(VI) in a later stage of the reaction.^12,27,31^Figure 2 shows the formation of PSO and PSO2 over time as monitored by FTIR spectroscopy. As the reaction progressed, a peak at ca. 1030 cm^–1^ appeared within the first 15 min of the reaction; this peak belonged to the stretching mode of the SO bond and was characteristic of the presence of sulfoxide groups. Then, after 2 h of reaction, a peak characteristic of SO_2_ stretching at ca. 1122 cm^–1^ appeared, indicating the formation of sulfone groups. After 48 h of reaction, only the SO_2_ peak had a detectable signal. The results suggest the concomitant formation of PSO and PSO2. However, the vibration of the C–S bonds was very weak in FTIR spectra, and it was challenging to ascertain the complete oxidation of the parent sulfide using this method.

Using ^13^C NMR spectroscopy, it was possible to track the progress of the reaction by following the signal from the carbon atoms in α and β of the sulfur centers in the EDDT-DAA PSR and analyzing the conversion of the PSR network. Figure 3a shows the NMR spectra of EDDT-DAA PSR NPs after different reaction times. During the course of the reaction, there were clear changes in the chemical environment within the polymer, with the β-carbons shifting upfield while the α-carbons shifted downfield. Figure 3 shows that all of the S(II) centers were rapidly converted in a mixture of S(IV) and S(VI), as a result of the autocatalytic nature of the reaction with H_2_O_2_.^22,32,33^ After 30 min of the reaction, all of the sulfur(II) atoms of the parent PSR were converted in a mixture of ca. 77 mol % PSO and 23 mol % PSO2. As the reaction progressed, all of the PSO was converted into PSO2 after 48 h. Consequently, a strong oxidizing agent, such as H_2_O_2_, even in a dispersed medium, is not adopted for producing pure PSO. Milder reaction conditions were necessary to isolate the pure PSO NPs.

**Figure 4:**
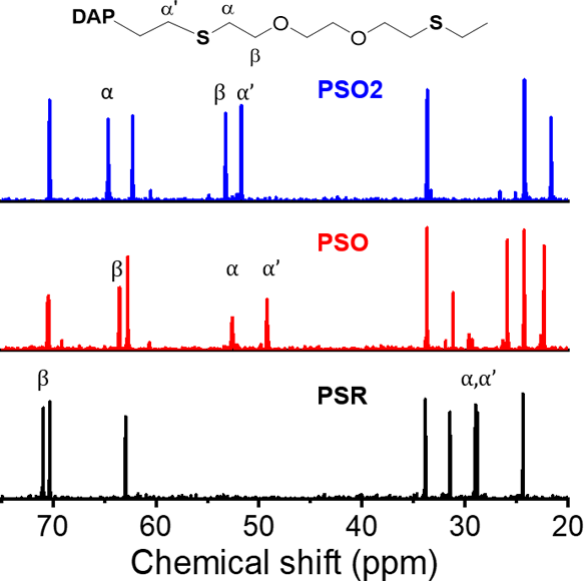
Selective and controlled reaction with different oxidants. ^13^C NMR spectra of pure polysulfide (black), polysulfoxide (red), and polysulfone (blue). Polysulfoxide was produced by the oxidation of the parent PSR with *t*-butyl peroxide, and polysulfone with hydrogen peroxide.

To promote the formation of PSO, the aqueous suspensions of PSR NPs of EDDT-DAA were reacted using *t*-BuOOH instead of H_2_O_2_ as the oxidizing agent, since alkyl peroxides provide milder reaction conditions than hydrogen peroxide.^34,35^Figure 4 shows the variation of the oxidation state of the PSR NPs after 24 h of the reaction with H_2_O_2_ and *t*-BuOOH. The ^13^C spectra of the polymer NPs obtained after 24 h of the reaction with either H_2_O_2_ or *t*-BuOOH clearly showed that the composition of the polymer changed during the reaction. When treated with H_2_O_2_, the sample transitioned from PSR to PSO and then to PSO2, and after 24 h of the reaction, only PSO2 was visible on the NMR spectra. After the same reaction time, the samples reacted with *t*-BuOOH displayed PSO groups, characterized by the carbons in the vicinity of sulfur at 49.2 and 52.6 ppm. The results obtained both by ^13^C NMR (Figure 4) and by FTIR spectroscopies (Figure S1) confirmed this result. Using a mild oxidant such as *t*-BuOOH, we successfully realized the selective synthesis of the intermediate-oxidation-state PSO. The results showed that using the right oxidizing agent could lead to the quantitative conversion of PSR in either PSO or PSO2.

The selectivity of the reaction observed can be attributed to the redox potential of the oxidant^36^ and to the fact that the transition from PSO to PSO2 requires a stronger oxidizing power than the potential involved in the formation of PSO from PSR.^34,37,38^ Secondary radicals such as alkyl peroxides are less reactive and thus more selective, offering milder options to control redox processes.^34,35,39−41^ The present results show that the alkyl peroxide was not a strong enough oxidant to oxidize PSO to PSO2. Moreover, experiments carried out for 70 days with samples reacted with *t*-BuOOH showed no formation of PSO2 by FTIR and ^13^C NMR spectroscopies.

**Figure 5:**
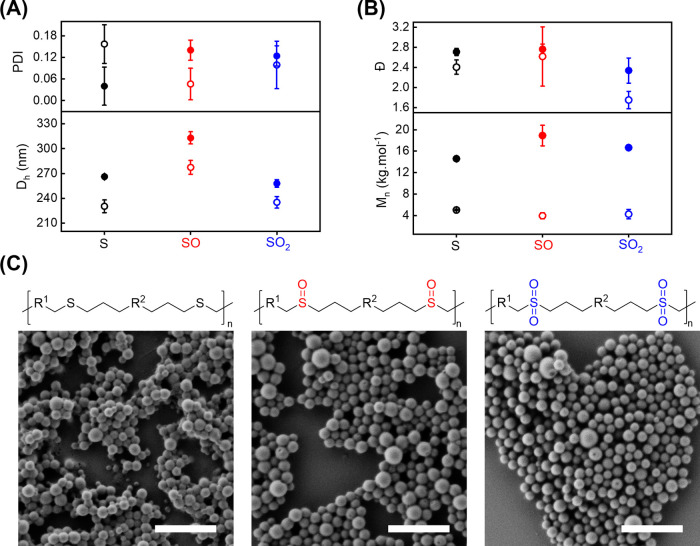
Colloidal stability and integrity of the polymer chains and NPs during oxidation of the parent polysulfide produced by the photoinitiated thiol-ene emulsion polymerization of 2,2′-(ethylenedioxy)diethanethiol and diallyl phthalate (solid symbols) or diallyl adipate (open symbols) in their original polysulfide (black), polysulfoxide (red), or polysulfone (blue) oxidation state. (A) Hydrodynamic size and the polydispersity index of the NPs measured by DLS indicating an increase in size for the polysulfoxide NPs. (B) *M*_n_ and dispersity of the polymer chains with different degrees of oxidation measured by GPC in DMF. (C) Scanning electronic microscopy images of the NPs. These NPs were cross-linked using triallyl phthalate to prevent the deformation of the soft and rubbery non-cross-linked NPs. Scale bars in SEM images stand for 500 nm.

A common challenge observed during the oxidation of PSRs is their overoxidation leading to the depolymerization of the polymer chains or the degradation of colloids.^13,23^ In addition, when the reaction occurs in suspension, the addition of a reagent to the continuous phase has the potential to destabilize the colloids.^42^Figure 5 shows that the colloidal stability and the polymer integrity were preserved during the oxidation of PSR NPs to PSO and PSO2 NPs. Characterization of the *M*_n_ of the polymers (EDDT-DAA and EDDT-DAP) by GPC after 24 h of oxidation with either H_2_O_2_ or *t*-BuOOH displayed no variation in *M*_n_ that would indicate the occurrence of chain-scission events during the oxidation reaction (Figure S2).

**Figure 6:**
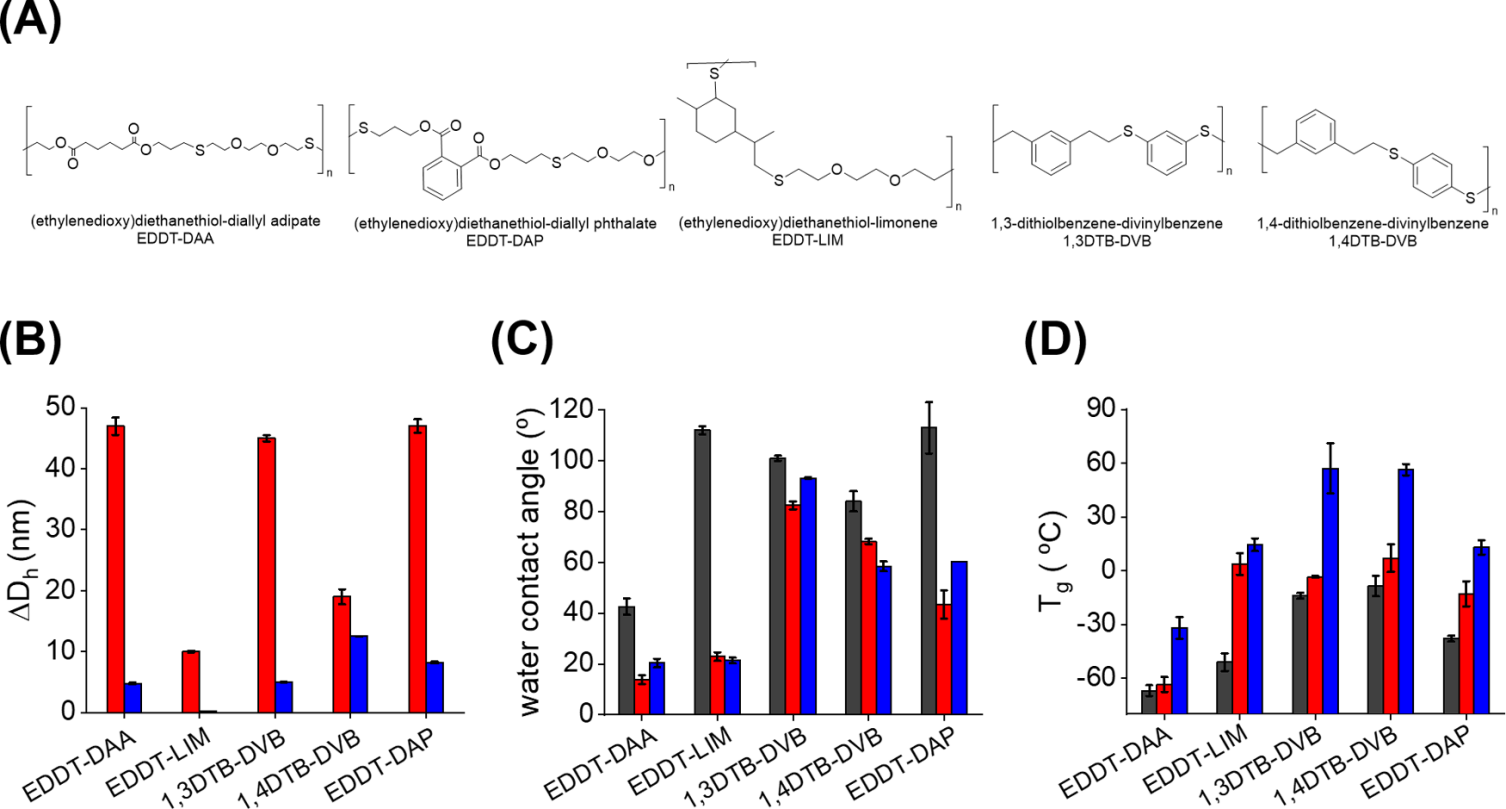
Properties of a library of sulfur-containing polymers. (A) Structures of the thiol-ene couple used as parent polysulfides. (B) Variation of the hydrodynamic diameter of the nanoparticles in suspension, (C) water contact angles, and (D) glass transition temperatures of the parent polysulfides (black) and their oxidized derivative polysulfoxides (red) and polysulfones (blue).

The size of the NPs measured by DLS during the reaction also did not show signs of degradation. First, during the formation of the PSO NPs, limited swelling (ca. 20%) of the NPs was observed. This phenomenon resulted from an increase in the hydrophilicity of the network, leading to an increased influx of the medium into the core of the NPs.^30,43^ However, when the NPs were fully oxidized to PSO2 NPs, the size of the oxidized NPs was similar to the size of the parent PSR NPs, suggesting that the PSO2 NPs were either less hydrophilic than the PSO NPs or that the interchain interactions were stronger in the PSO2 NPs than in the PSO NPs. We observed the same behavior during the oxidation of every parent PSR NP (Figure 6a,b). Furthermore, the SEM images of the dry NPs showed no difference in the size of the NPs containing sulfur atoms with different oxidation states (Figures 5 and S3). This observation indicates that the increase in size observed for the PSO NPs in suspension was likely the result of stronger intermolecular interactions with water molecules in PSOs than in the PSO2 case, leading to the preferential swelling of PSO in keeping with the higher H-bonding of SO derivatives observed previously.^44−46^ The analysis of the NPs by SEM before and after oxidation did not show any detrimental effects of the reaction on the size nor the morphology of the NPs, corroborating the results obtained by GPC.

Different parent PSR NPs were prepared and oxidized with both H_2_O_2_ and *t*-BuOOH (Figure 6a). Each case resulted in NPs with preserved colloidal stability and degree of polymerization, with a controlled oxidation state of the sulfur atoms, from S(II) to either fully S(IV) or fully S(VI) (Figure S1). For every parent PSR NP used, similar swelling (ca. 20%) of the NPs was observed after oxidation to PSO but the swelling decreased to only ca. 3% for PSO2 (Figure 6b), as observed with EDDT-DAA (Figure 5). Although the oxidation reaction of the PSR network did not significantly influence the size and stability of the polymer NPs in suspension, the properties of the resulting oxidized polymers were affected. Films cast from the polymers after oxidation showed an increase in hydrophilicity for the oxidized derivatives (Figures 6c and S4), resulting in a lower water contact angle on the films prepared with PSO and PSO2 in comparison to those made with PSR. However, the difference between the wetting of PSO and PSO2 was limited. The increased hydrophilicity of the oxidized derivatives was due to dipolar interactions between the polymer chains and the solvent and could potentially be harnessed in the design of biomaterials for in vivo applications.^16,47−49^

The oxidation state of the sulfur centers also influenced the thermomechanical properties of the polymers. As the oxidation state increased, the glass transition temperatures (*T*_g_) of the polymer also increased (Figure 6d). In the case of the parent PSRs containing aromatic moieties, the polymers prepared with the cyclic monomers dithiol benzene and divinyl benzene yielded polymers with higher *T*_g_ in comparison to the polymer prepared with aliphatic structures due to the strong interchain interactions created by π–π stacking. The oxidation reaction of PSR to PSO resulted in only a limited increase in *T*_g_. However, after the formation of fully oxidized PSO2, the *T*_g_ steeply increased, possibly due to the loss in flexibility caused by the interchain dipolar interactions between the sulfone groups (Figure S5).^14,15^ Furthermore, the oxidation state of the sulfur atoms also influenced the thermal degradation of the polymers (Figure S6 and Table S2). The thermal stability of the network decreased upon the formation of PSOs. For most polymers, the onset of degradation decreased by ca. 120 °C but only by ca. 10–20 °C for the polymers prepared with the aromatic dithiol. In general, the formation of SO groups in the polymer backbone destabilized the polymer network and led to early decomposition. This effect likely occurred due to a Pummerer elimination in the sulfoxide-containing polymers, in which the rearrangement of the SO groups within the chains led to the rapid degradation of the polymer scaffold.^15,27,50^ As the oxidation increased and the PSO2 polymers were fully formed, the thermal stability increased. While some poly(olefin sulfone)s can show self-immolative properties,^51,52^ the backbone of the PSO2s formed by thiol-ene addition followed by oxidation yielded thermally stable polymer materials. However, the thermal stability of PSO2s was the highest in comparison to PSOs and similar to PSRs.

The characterization of the polymers produced from the different parent PSRs showed that the oxidation reaction increased the polarity of the polymer backbone, regardless of the parent polysulfide backbone without adverse effects on the colloidal suspension. However, only the presence of SO_2_ groups increased the thermal stability of the polymer, while the intermediate SO groups induced a decrease in thermal stability but provided the polymer with higher wettability. The production of well-defined PSRs, PSOs, and PSO2s with distinct physicochemical properties paves the way to use these new materials in an array of applications, from control drug delivery systems to smart coatings.

## Conclusions

Controlled oxidation of parent polysulfide nanoparticles in waterborne suspensions yielded either pure polysulfoxides or pure polysulfone latexes. Photoinitiated thiol-ene emulsion polymerization was used to generate a library of parent polysulfide nanoparticles. Then, employing two distinct oxidants, *tert*-butyl hydroperoxide and hydrogen peroxide, the reactions were effective in both controlling the oxidation state of the sulfur atoms and retaining the colloidal stability of the nanoparticles and the integrity of the polymer chains within the nanoparticles. Oxidation of polysulfides by H_2_O_2_ led to the formation of polysulfones after a 24 h long reaction time. Kinetic studies of oxidation via H_2_O_2_ showed the coexistence of sulfone and sulfoxide throughout the reaction until full conversion into polysulfone, highlighting the need for a method based on the selective control of the reaction to produce pure polysulfoxide. Conversely, the reaction with *tert*-butyl peroxide, a milder oxidant, allowed for the selective production of polysulfoxides. In addition, the oxidation process was robust and versatile, yielding fully oxidized polysulfoxide or polysulfone using a variety of parent polysulfides. With control over the conversion of the pure oxidized species, we analyzed the unique physicochemical properties of the polysulfoxides and polysulfones. The polysulfones displayed better thermal stability and a higher glass transition temperature than the parent polysulfides and polysulfoxides, while the polysulfoxides displayed higher hydrophilicity than the polysulfides and polysulfones. The synthetic process introduced here provides a robust synthetic platform to control the design of sulfur-containing polymers with a wide range of finely tuned physicochemical properties, and the resulting materials can find applications as controlled release systems with enhanced “stealth effect” or as the next generation of packaging materials.

## Supporting Information Available

Additional characterization of the nanoparticles and the respective polymers (FTIR and ^13^C NMR spectra, GPC, SEMm DLS, water contact angles, DSC, and TGA) (PDF)
