# Supplementary material for: Selective Oxidation of Polysulfide Latexes to Produce Polysulfoxide and Polysulfone in a Waterborne Environment
Source: Macromolecules. 2021 Apr 8;54(8):3659–67. doi: 10.1021/acs.macromol.1c00382 (PMC8161668; doi:10.1021/acs.macromol.1c00382)
Supplement: Supplementary file 1 — ma1c00382_si_001.pdf [file ma1c00382_si_001.pdf]

Supporting information

Selective oxidation of polysulfide latexes to produce polysulfoxide and polysulfone in a waterborne environment

Lorena Infante Teixeira, Katharina Landfester, Héloïse Thérien-Aubin**

Max Planck Institute for Polymer Research, Ackermannweg 10, 55128 Mainz (Germany).

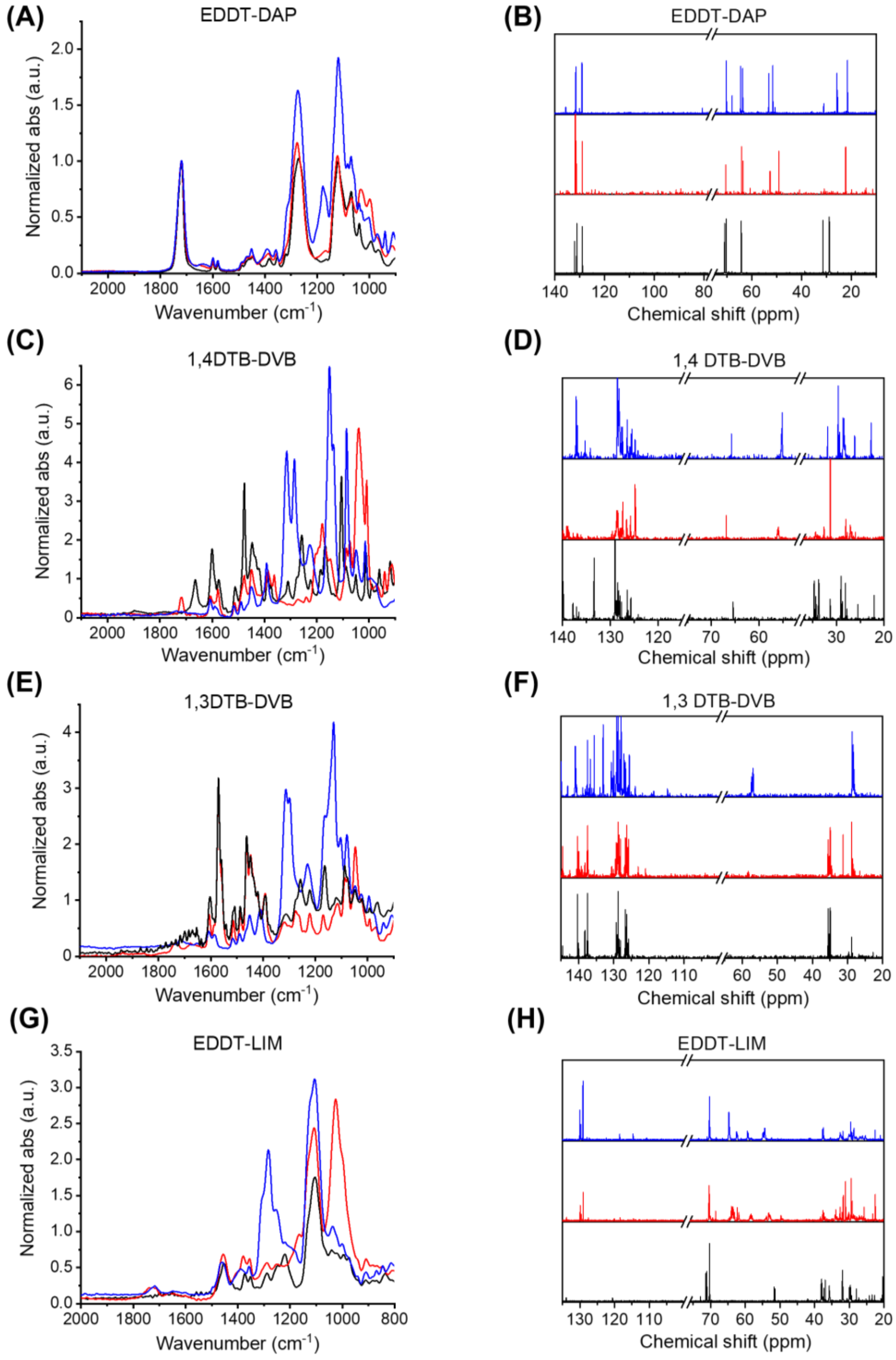

Figure S1. Characterization of the selective oxidation of polysulfides by H_2O_2 and t-BuOOH. FTIR and ^{13}C-NMR spectra taken from each polysulfide (**black**), and their oxidated derivatives, polysulfoxide (**red**) and polysulfone (**blue**). Spectra were gathered in a) for EDDT-LIM, b) EDDT-DAP, c) 1,3DTB-DVB, and d) for 1,4DTB-DVB group of sulfur-containing polymers.

Table S1. Characterization of the parent polysulfides synthetized. Average molecular weight and dispersion coefficient were obtained by GPC. The conversion was measured by ^{1}H-NMR spectroscopy from the integration of remaining allyl/vinyl protons.

HS/C=C	Conversion (%)	*M*n (kg.mol^{-1})	*Đ*
EDDT-DAA	> 99[a]	5020 (125)[c]	2.4 (0.1)
EDDT-DAP	> 99[a]	15000 (200)	2.8 (0.1)
EDDT-LIM	97[a]	5800 (1000)	2.7 (0.3)
1,3DTB-DVB	91[a]	2330 (20)	7.1 (0.3)
1,4DTB-DVB[2]	90[b]	2300 (50)	2.1 (0.4)

[a] Measured in $CDCl_3$
[b] Measured in d_6-DMSO
[c] The numbers in parenthesis are the standard deviations

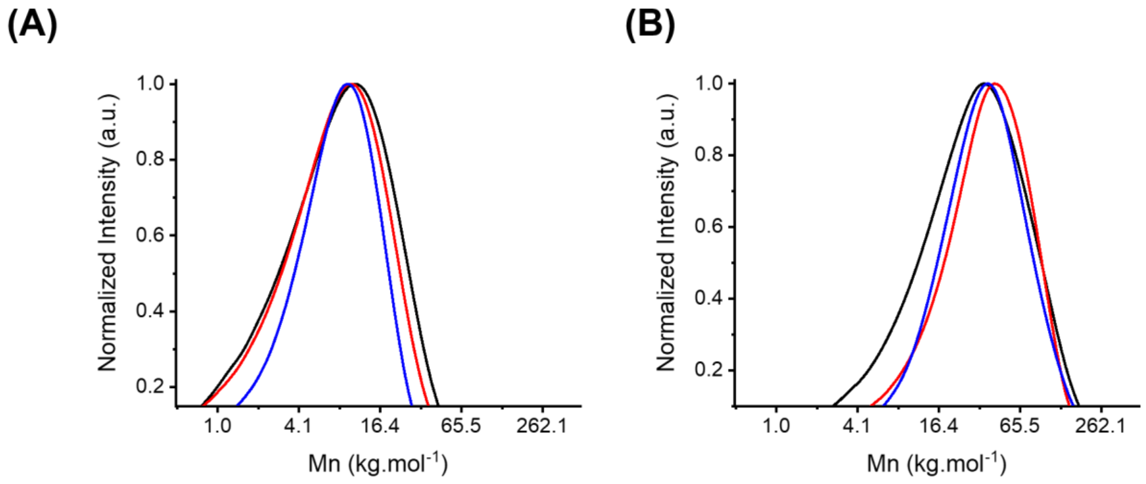

Figure S2. GPC traces of polysulfide (black), polysulfoxide (red), and polysulfone (blue) of (a) EDDT-DAA, and (b) EDDT-DAP systems.

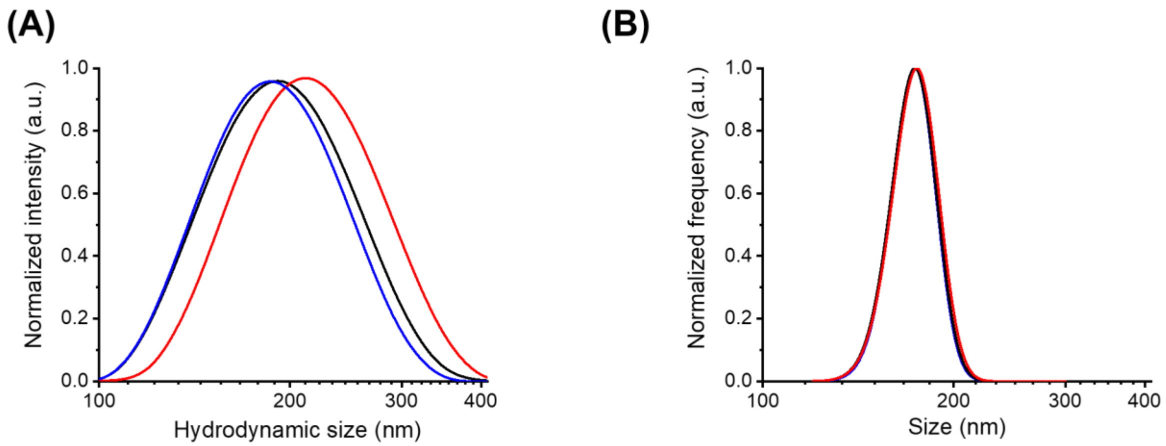

Figure S3. Comparison of the sizes of TAP-EDDT cross-linked NPs at different degrees of oxidation measured a) in suspension by DLS, and b) as dried particles by SEM imaging for the polysulfide (black), polysulfoxide (red), and polysulfone (blue). The data indicate a preferential swelling of PSO NPs when in suspensions (a), which was not observed in dried state (b).

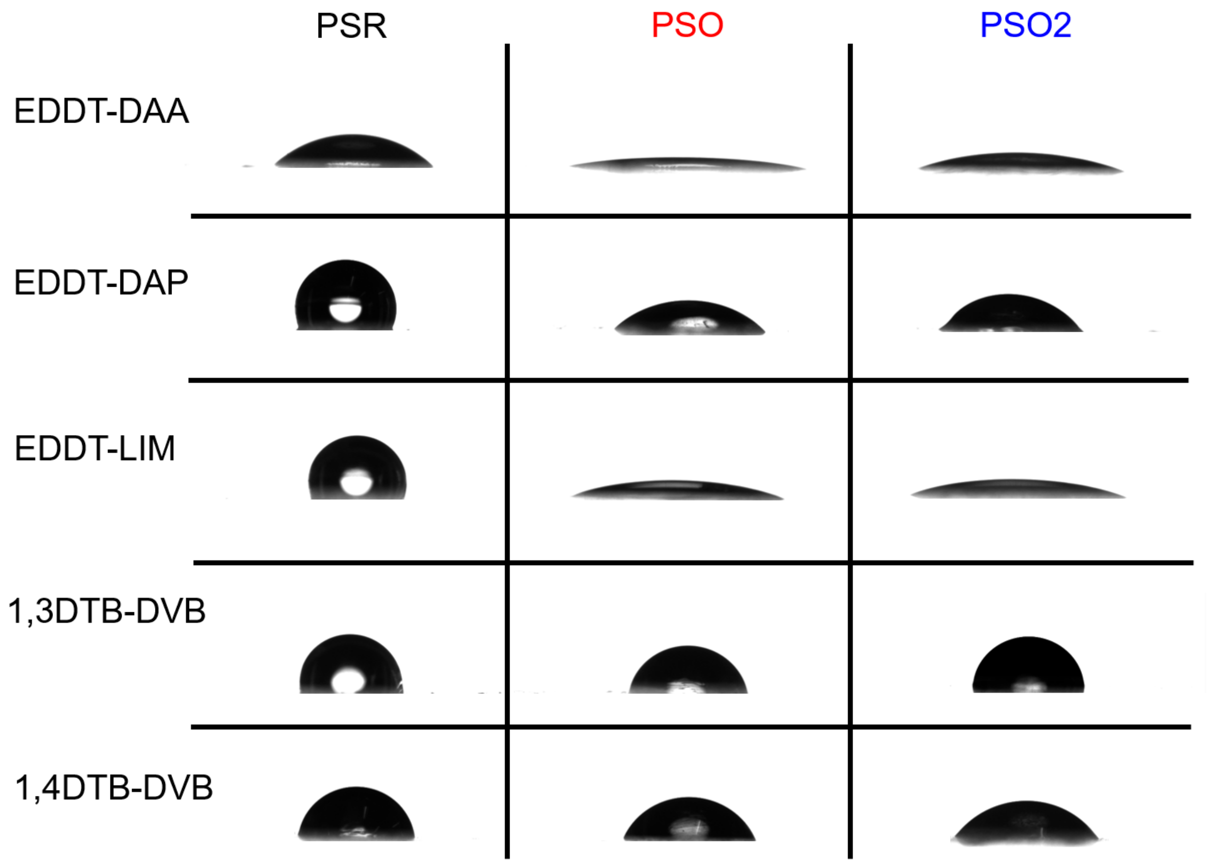

Figure S4. Photography of water droplets on the surface of glass slides covered with film of polymers. The contact angle of the water droplets, i.e. hydrophilicity, on the surfaces of the films decrease after oxidation, and was the lowest for the polysulfoxides.

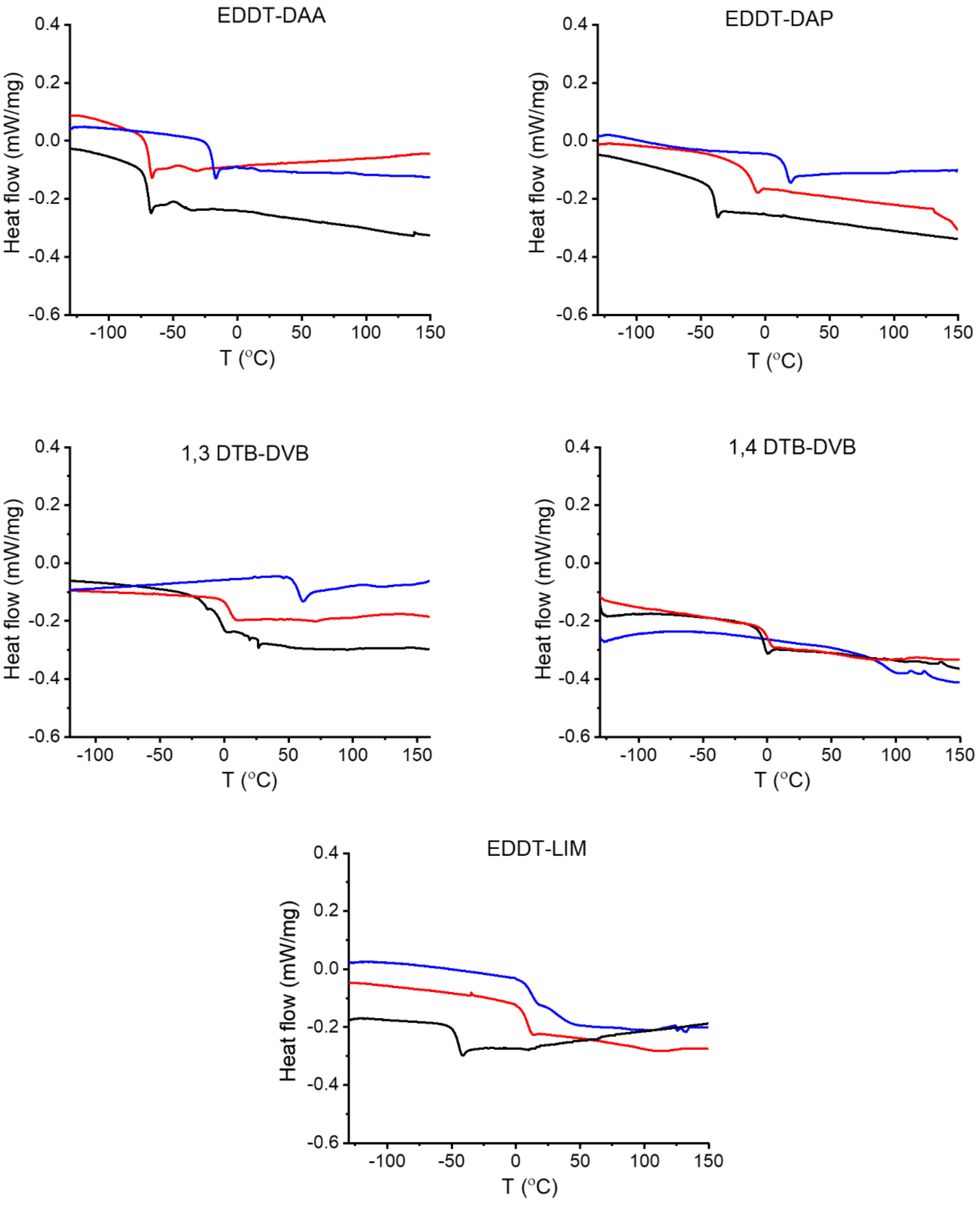

Figure S5. DSC traces of polysulfide (black), polysulfoxide (red), and polysulfone (blue) of all systems.

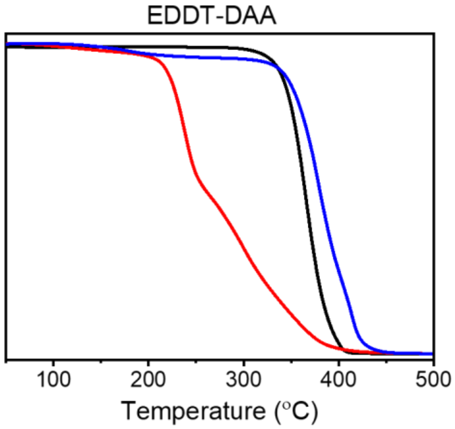

Figure S6. TGA curves of polysulfide (**black**), polysulfoxide (**red**), and polysulfone (**blue**) of EDDT-DAA system.

Table S2. Degradation temperature of the polymers before and after oxidation measured by TGA.

HS/C=C	PSR	PSO	PSO2
EDDT-DAA	368 (3)	244 (3)	382 (2)
EDDT-DAP	362 (2)	246	340 (1)
EDDT-LIM	355 (5)	236 (6)/ 295 (9)	353 (2)
1,3DTB-DVB	350 (14)	333 (8)	340 (3)
1,4DTB-DVB	335 (5)	327 (4)	347 (3)

The numbers in parenthesis are the standard deviations
